# The lead time and geographical variations of Baidu Search Index in the early warning of COVID-19

**DOI:** 10.1038/s41598-023-41939-z

**Published:** 2023-09-07

**Authors:** Yuhua Ruan, Tengda Huang, Wanwan Zhou, Jinhui Zhu, Qiuyu Liang, Lixian Zhong, Xiaofen Tang, Lu Liu, Shiwen Chen, Yihong Xie

**Affiliations:** 1 State Key Laboratory of Infectious Disease Prevention and Control (SKLID), National Center for AIDS/STD Control and Prevention (NCAIDS), Chinese Center for Disease Control and Prevention (China CDC), Collaborative Innovation Center for Diagnosis and Treatment of Infectious Diseases, Beijing, China; 2 Department of Epidemiology and Biostatistics, Guangxi Medical University, Nanning, China; 3 Guangxi Key Laboratory of Major Infectious Disease Prevention Control and Biosafety Emergency Response, Guangxi Center for Disease Control and Prevention, Nanning, China; 4 Department of Health Management, The People’s Hospital of Guangxi Zhuang Autonomous Region & Research Center of Health Management, Guangxi Academy of Medical Sciences, Nanning, China; 5 Guangxi Colleges and Universities Key Laboratory of Prevention and Control of Highly Prevalent Diseases, Guangxi Medical University, Nanning, China

**Keywords:** Diseases, Medical research

## Abstract

Internet search data was a useful tool in the pre-warning of COVID-19. However, the lead time and indicators may change over time and space with the new variants appear and massive nucleic acid testing. Since Omicron appeared in late 2021, we collected the daily number of cases and Baidu Search Index (BSI) of seven search terms from 1 January to 30 April, 2022 in 12 provinces/prefectures to explore the variation in China. Two search peaks of “COVID-19 epidemic”, “Novel Coronavirus” and “COVID-19” can be observed. One in January, which showed 3 days lead time in Henan and Tianjin. Another on early March, which occurred 0–28 days ahead of the local epidemic but the lead time had spatial variation. It was 4 weeks in Shanghai, 2 weeks in Henan and 5–8 days in Jilin Province, Jilin and Changchun Prefecture. But it was only 1–3 days in Tianjin, Quanzhou Prefecture, Fujian Province and 0 day in Shenzhen, Shandong Province, Qingdao and Yanbian Prefecture. The BSI was high correlated (r_s_:0.70–0.93) to the number of cases with consistent epidemiological change trend. The lead time of BSI had spatial and temporal variation and was close related to the strength of nucleic acid testing. The case detection ability should be strengthened when perceiving BSI increase.

## Introduction

As of 10 August 2022, the ongoing novel coronavirus infection (COVID-19) pandemic has affected all countries and territories with more than 584 million confirmed cases and over 6.4 million deaths have been reported globally^1^. The etiological agent of COVID-19 is belong to the coronavirus family with a high rate of mutation^2^. The emergence of variants has posed an increased risk to global public health and prompted the identification of specific variants in late 2020. Thus far, the variants of concern are Alpha, Beta, Gamma, Delta, and Omicron^3^. The integrated control measures including lockdown, confinement, keeping social distance, hygiene, wearing masks, vaccination, regular nucleic acid testing, etc., but the implementation strength was vary by region. With the scientific research progress of COVID-19, more precise and differentiated epidemic control strategies was implemented. And the timeliness of diagnosis had obviously improved after launched massive nucleic acid testing^4^, which screening up all the residents in areas of high risk of infection to detect potential infection source earlier, cut transmission and achieve dynamic zero-COVID-19 at the social level. Early detect the epidemic in a certain area was critical to control the further spread and to support the public health authority making decision. The previous studies focused on the early warning of COVID-19 mainly used wastewater-based viral RNA surveillance^5–8^ and web-based search query data^9–17^. Sewage surveillance of SARS-CoV-2 RNA has been suggested as an early warning tool for alerting the circulation in communities^5–8^. However, there are many challenges in sewage-surveillance, such as sampling sites, sampling strategies, detection and quantification methods of SARS-CoV-2, the high cost, etc.^18,19^. The web-based search query data could serve as a convenient indicator for predicting infectious disease outbreak^20–23^ and the epidemic of COVID-19^9–17^. With internet search data and social media data, the epidemic waves of COVID-19 could be detected 10–14 days earlier in China^12^, 1–2 weeks earlier in US and Canada^16^, 2–3 weeks earlier in India^15^, 11 days earlier in Spain^11^. However, only limited studies focused on the early warning of COVID-19 using internet search data. And they were mostly conducted in the first half year of 2020, when there was still insufficient knowledge about this emerging disease and before the emergence of more contagious but less fatal variants e.g., Omicron^24^. The attention of internet user may be affected by the epidemic intensity, mutant strain and specific public health actions. In addition, the pre-warning time may have spatial and temporal variation as it is closely related to the medical resource allocation and the timeliness of diagnosis. Our recent research in Wuhan, Hubei (excluded Wuhan) and China (excluded Hubei) had yielded an increased awareness of this diversity^17^. With the implement of massive nucleic acid testing, the effectiveness and the indicators of internet search data in COVID-19 pre-warning may change over time and space. Yet there was no study focused on the spatial and temporal variation since the new variants appeared and the implementation of massive nucleic acid testing.

On 9 November, 2021, a more contagious variant of COVID-19 B.1.1.529 (named Omicron on 26 November 2021by WHO) was first detected in South Africa from a case sample^25^. In the following few months, this mutant replaced Delta and became the absolute dominant variant of COVID-19 globally^24^. In China, the first Omicron variant imported case was detected from an asymptomatic international traveler in Tianjin City on 13 December 2021^26^. And the first two local Omicron infection cases in China were also detected in Tianjin on 8 January 2022^27^. Then several severe epidemics dominated by Omicron occurred in China in first four months of 2022, including four provinces (Henan, Jilin, Shandong, Fujian province), two municipalities (Shanghai, Tianjin City) and the special economic zones (Shenzhen City). As the epidemic intensity, economic level, medical resource allocations and the implementation strength of massive nucleic acid testing actions were different in these areas, the public attention and the lead time of Baidu Search Index may also different in different area and different phase. Here we explore the geographical and temporal variations of Baidu Search Index in the early warning of COVID-19 and identify impacting factors. The findings may provide scientific evidences for the early detected of disease outbreak and improve the disease surveillance system using internet search data, especially for the emerging and re-emerging infectious diseases.

## Methods

### Study areas

In early 2022, with the occurrence of Omicron, several provinces/municipalities in China reported a relatively large scale of COVID-19 epidemic between 1January to 30 April, 2022, which including four provinces (Henan, Jilin, Shandong and Fujian Province), two municipalities (Shanghai and Tianjin City) and one special economic zone (Shenzhen City). In this study, we mainly focus on these seven provinces/municipalities/special economic zones. Considering Shandong, Henan, Fujian and Jilin Province have multiple administrated prefectures and their population size were 102, 98.83 million, 41.87 million and 23.75 million, respectively, in 2020, while the epidemic mainly confined to a few prefectures of these provinces. For comparison, we focused on both the whole province and the specific epidemic prefectures. The main epidemic prefectures were Jilin Prefecture, Changchun Prefecture and Yanbian Korean Autonomous Prefecture in Jilin Province, Qingdao Prefecture in Shandong Province and Quanzhou Prefecture in Fujian Province.

### Data source

The daily new confirmed cases and asymptomatic cases from 1 January to 30 April, 2022 was obtained from the National Health Commission (http://www.nhc.gov.cn/)^28^. More specific information and control measure, and the dominated variant in each epidemic was obtained from the website of local Health Commission of each study area. The internet search data we focused on Baidu, which is the most popular search engine in China and with more than 90% of Chinese internet users^29^. The daily search query data of Baidu Search Index for each search term (including PC + Mobile) was achieved from the Baidu Index Platform^30^. The search terms used in achieving the Baidu search volume were those highly related to COVID-19. The specific11 search terms were “Novel coronavirus (新型冠状病毒) or (新冠病毒) or (冠状病毒)”, “Omicron (奥密克戎)”, “Delta (德尔塔)”, “COVID-19 (新型冠状病毒肺炎) or (新冠肺炎) or (新冠) or (冠状病毒肺炎)”, “Pneumonia (肺炎)”, “COVID-19 epidemic (新型冠状病毒肺炎疫情) or (新冠肺炎疫情) or (疫情)”, “Mask (口罩, including N95)”, “Nucleic acid (核酸)”, “COVID-19 vaccine (新冠疫苗)”, “Antigen (抗原)” and “Asymptomatic patient (无症状感染者)”. Similar search terms for different expressions were subsequently combined. “Omicron” and “Delta” was combined to “Novel Coronavirus”, “Pneumonia” was combined to “COVID-19”, and “Antigen” was combined to “COVID-19 vaccine”. Finally, the Baidu Search Index of seven combined search terms ie. “Novel Coronavirus”, “COVID-19”, “COVID-19 epidemic”, “Mask”, “Nucleic acid”, “COVID-19 vaccine”, and “Asymptomatic patient” were used in the data analysis. For comparison, the search volume was weighted by the number of populations. As the publication delayed, we used the population data in year 2020 and achieved from the local Statistical Yearbook of each study area.

### Statistical analysis

**Figure 1:**
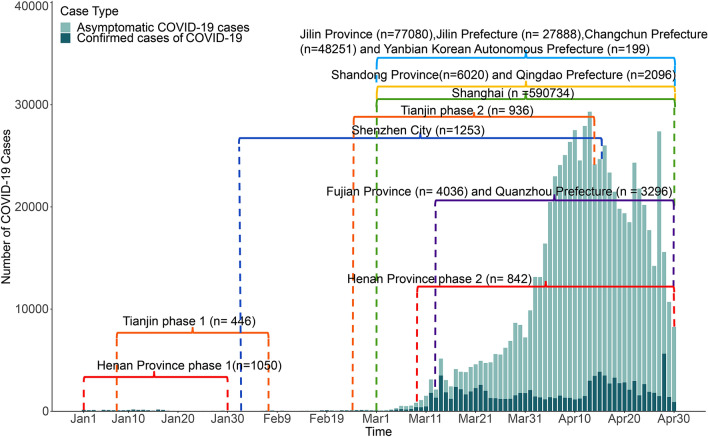
The main epidemic of COVID-19 in China from 1 January to 30 April, 2022. (n showed the total number of new confirmed cases and asymptomatic cases in each study area in the main epidemic period of concerned).

The epidemic curve was used to describe the temporal distribution of the daily new confirmed cases and asymptomatic cases and the Baidu Search Index (per million populations) from 1 January to 30 April, 2022, and explore the indicators that can be used in the early warning of COVID-19 epidemic and examine the pre-warning time. Spearman correlation was used to analyze the correlation between the number of daily cases (new confirmed and asymptomatic) and the Baidu Search Index with different lead time. As the epidemic period and the lead time in each study areas were different, the data used in Spearman correlation analysis were only focus on 14 days before and after each main epidemic (the epidemic period of concerned in each study area was showed in Fig. 1) and the results were presented with 8 days, 15 days or 30 days lead time. Weak, moderate and high correlation was classified according to the Spearman’s correlation coefficient (r_s_) value < 0.40, 0.40–0.7, > 0.7, respectively. Considering the high correlation between the search volume of each indicator, those indicators that significant associated with the number of cases were used to conduct a factor analysis, with maximum variance rotation method and the number of common factors was judged by initial eigenvalues > 1. As the daily incidence of COVID-19 was over-dispersed, a negative binomial regression model was built with the dependent variable as the daily number of cases weighted by population and independent variable was the factor scores. Smallest Akaike's information criterion (AIC) indicated the best model fit in different lead time. The statistics analysis was performed using R version 4.0.2 software (R Foundation for Statistical Computing) with “MASS”, “psych” and “epicalc” package. The statistical significance level was set as 0.05.

## Results

### The main epidemic of COVID-19 in China from January 1 to April 30, 2022

Figure 1 showed the main epidemic period on concerned in each study areas and the corresponding number of daily new confirmed and asymptomatic cases. In January, a local epidemic occurred in Henan Province (1 January to 30 January, 2022) and Tianjin City (8 January to 7 February, 2022) with 1050 and 446 cases reported, respectively. A second peak can be observed in Henan Province (9 March to 30 April, 2022) and Tianjin City (24 February to 13 April, 2022), which reported 842 and 936 cases, respectively. There were only some sporadic Omicron related cases in Shenzhen City in January (20 cases) and early February (17 cases), since then the cases consecutive increased with 1236 cases reported from 12 February to 22 April, 2022. There were also a few sporadic Omicron related cases in Shanghai City in January (4 cases) and February (9 cases). However, the most severe epidemic areas and periods were Shanghai City (590,734 cases), Jilin Province (77,080 cases) and Shandong province (6,020 cases), where the epidemic last from early March to the end of April. Except the epidemic in Henan Province (phase 1) in January was dominated by Delta variants, all the others were dominated by Omicron variants, and most of cases were detected through massive nucleic acid testing without symptoms.

### Lead time of Baidu Search Index in the early warning of COVID-19 in each epidemic area in China from January 1 to 30 April, 2022

**Figure 2:**
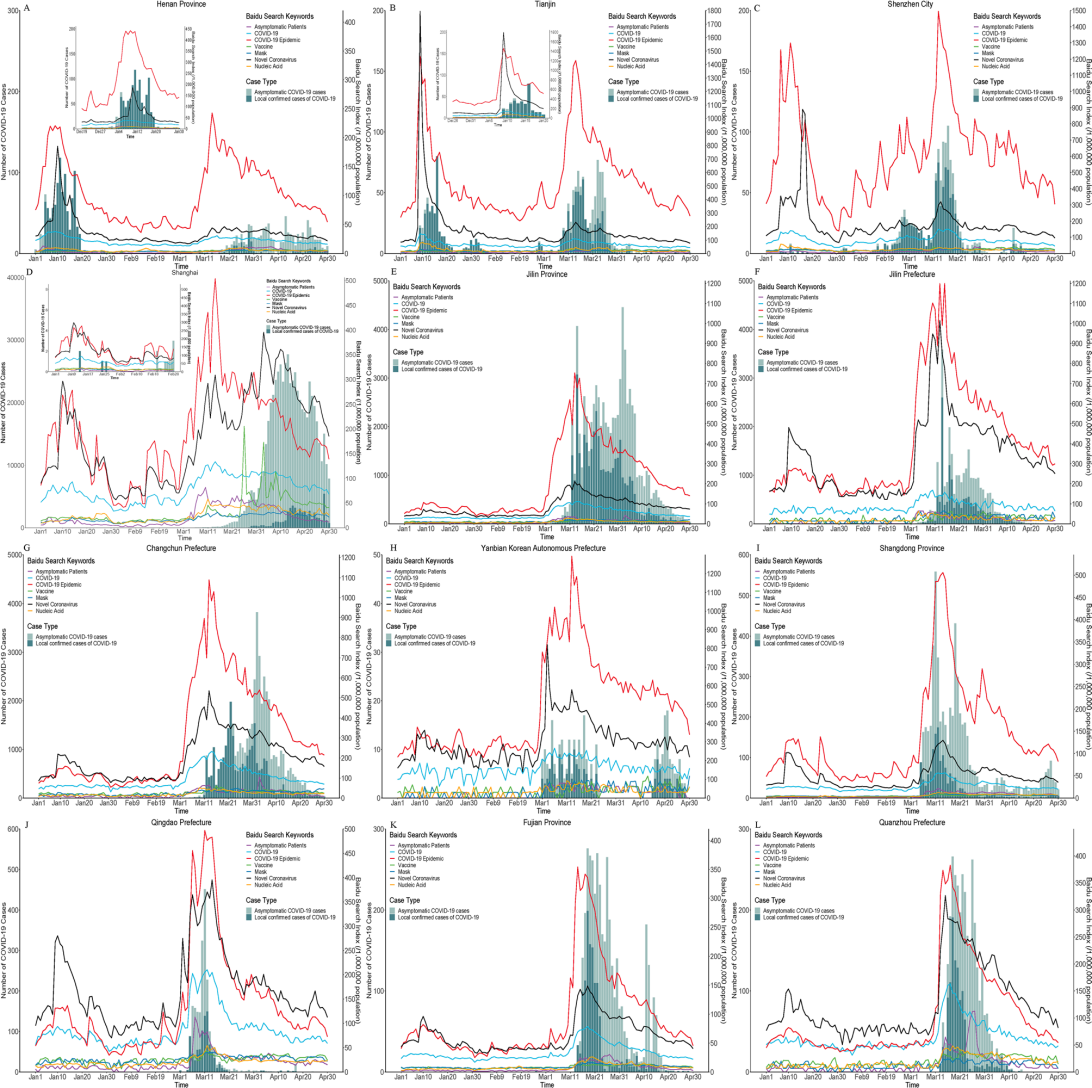
The epidemic curve of the number of daily new confirmed and asymptomatic cases and the Baidu Search Index volume (per 1,000,000 population) in each study areas from 1 January to 30 April, 2022. (Note: As the first peaks of epidemic in Henan Province and Tianjin were occurred in early January 2020, a small graph was drawn to show the dynamic change of Baidu search volume 14 days before the epidemic in (**A**,**B**).

In January, the search volume of “COVID-19 epidemic” and “Novel Coronavirus” suddenly increased on 9 January and peak on 10–12 January in all the study areas. In Henan Province and Tianjin City, where had local epidemic, the search volume of “COVID-19 epidemic” and “Novel Coronavirus” increased 3 days ahead of the increased of cases (Fig. 2A,B, Table 1). The search peak of “COVID-19 epidemic” was higher than “Novel Coronavirus” in Henan Province (Fig. 2A) and Shenzhen City (Fig. 2C). A similar search peak of “COVID-19 epidemic” and “Novel Coronavirus” can be observed in Tianjin City (Fig. 2B) and Shanghai City (Fig. 2D). While in the other areas without cases reported, the search peak of “Novel Coronavirus” was higher than “COVID-19 epidemic” (Fig. 2F,G,J–L). In Tianjin and Shenzhen City, the search volume was as high as 1793 and 889 per 1,000,000 population, respectively (Fig. 2C,D). While the search peaks were only 100–400 per 1,000,000 population in the other provinces.

**Table 1:** The lead time of Baidu Search Index in each study area from 1 January to 30 April, 2022.

Study area	Period of concerned	Date of Baidu Search Index increased	Date of number of cases increased	Lead time
(days)				
Henan Province (phase 1)	2021.12.19–2022.02.12	2022.01.02	2022.01.03	1
Henan Province (phase 2)	2022.02.24–04.30	2022.03.06	2022.03.20	14
Tianjin City (phase 1)	2021.12.26–02.20	2021.01.06	2022.01.09	3
Tianjin City (phase 2)	2022.02.11–04.26	2022.03.07	2022.03.08	1
Shenzhen City^1^	2022.01.27–04.30	Fluctuated	Fluctuated	0
Jilin Province	2022.02.16–04.30	2022.03.02	2022.03.08	6
Yanbian Prefecture	2022.02.16–04.06	2022.03.01	2022.03.01	0
Jilin Prefecture	2022.02.18–4.30	2022.03.02	2022.03.08	6
Changchun Prefecture	2022.02.19–04.30	2022.03.02	2022.03.11	9
Shandong Province	2022.02.16–04.30	2022.03.05	2022.03.05	0
Qingdao Prefecture	2022.02.16–04.30	2022.03.05	2022.03.05	0
Shanghai City	2022.02.16–04.30	2022.02.14	2022.03.12	28
Fujian Province	2022.02.28–04.30	2022.03.10	2022.03.13	3
Quanzhou Prefecture	2022.02.28–04.28	2022.03.12	2022.03.13	1

*The main epidemic in Shenzhen City was from February 12 to April 22 while there were some sporadic cases in January and early February, the search volume was also fluctuated with the number of cases.

In February, the search volume of “COVID-19 epidemic” and “Novel Coronavirus” in Shenzhen City and Shanghai City were more fluctuated with few sporadic cases occurred. The search volume in Shenzhen increased on 6 February, which was 8 days ahead of the increased of cases. While the search volume in the other areas without cases reported was relatively stable and kept in low level in the whole February (Fig. 2).

On early March, an anomalous peak of Baidu Search Index of “COVID-19 epidemic”, “Novel Coronavirus” and “COVID-19” can be observed in all the study areas with the local epidemic occurred. But the lead time had spatial variation (Fig. 2, Table 1). The search volume increased 14–28 days ahead of the number of cases in a recently epidemic areas (Henan Province phase 2, Fig. 2A) and the most severe epidemic area (Shanghai City, Fig. 2D). There was only1 day lead time in Tianjin City (phrase 2) and 0 day lead time in Shenzhen City, but the search volume was obviously higher than the other study areas (Fig. 2B,C). There were 6–9 days lead time in Jilin province, Jilin Prefecture and Changchun Prefecture (Fig. 2E–G), but the search volume in the main epidemic areas (Jilin Prefecture and Changchun Prefecture) was significantly higher than that of the whole province. Only 1–3 days lead time can be observed in Fujian Province and Quanzhou Prefecture (Fig. 2K,L), where the epidemic was relatively small. However, 0 day lead time can be observed in Yanbian Korean Automous Prefecture (Fig. 2H), Shangdong Province (Fig. 2I) and Qingdao Prefecture (Fig. 2J).

From 1 January to 30 April, 2022, the search volume of “Mask”, “Nucleic acid”, “COVID-19 vaccine” and “Asymptomatic patient” were also increased with the number of cases increased. But the search volume kept in a very low level, mostly was less than 50 per 1,000,000 population and no obviously peak can be observed in most study areas.

### Spearman correlation between the number of daily reported cases and Baidu Search Index (per 1,000,000 populations) from January 1 to 30 April, 2022

Table 2 showed the Spearman correlation coefficients between the daily number of cases and Baidu Search Index (per million populations) with different lead time, which showed their change of epidemiological trend. The search volume of “Novel Coronavirus”, “COVID-19” and “COVID-19 epidemic” was correlated with the number of cases during the epidemic period of concerned in all study areas. The maximum association can be observed with different lead time, which was 14–28 days in Shanghai (r_s_:0.70–0.87), 11–14 days in Henan Province (phase 2, r_s_:0.72–0.80), 5–10 days in Jilin Province (r_s_:0.87–0.92), Jilin Prefecture (r_s_:0.74–0.93) and Changchun Prefecture (r_s_:0.79–0.91), while it can be observed in 0–3 days lead time in the other study areas.

**Table 2:** Spearman correlation between Baidu Search Index (/1,000,000 population) and the number of COVID-19 cases with different lead time in each study areas from 1 January to 30 April, 2022.

Study areas (periods of concerned)	Lead time (day)	Novel coronavirus	COVID-19	COVID-19 epidemic	Mask	Nucleic acid	COVID-19 vaccine	Asymptomatic patients
Henan Province (phase 1, 2021.12.19–02.12)	0	0.84	0.88	0.90	0.73	0.77	0.42	0.85
	1	0.80	0.87	0.84	0.73	0.75	0.47	0.76
	2	0.78	0.87	0.81	0.69	0.70	0.40	0.73
	3	0.70	0.86	0.77	0.71	0.66	0.34	0.66
	4	0.61	0.83	0.72	0.63	0.63	0.37**	0.68
	5	0.54	0.79	0.67	0.61	0.57	0.40	0.64
	6	0.47	0.72	0.60	0.60	0.49	0.42	0.59
	7	0.44	0.70	0.54	0.53	0.45	0.35**	0.51
	8	0.38	0.66	0.50	0.51	0.42	0.38	0.52
Henan Province (phase 2, 2022.02.24–04.30)	0	0.40	0.28**	0.20*	0.71	0.67	0.66	0.76
	1	0.44	0.3**	0.26**	0.72	0.70	0.61	0.75
	2	0.49	0.35	0.32**	0.73	0.69	0.60	0.72
	3	0.52	0.40	0.35	0.75	0.69	0.63	0.73
	4	0.54	0.47	0.42	0.74	0.69	0.62	0.71
	5	0.59	0.53	0.49	0.81	0.66	0.65	0.73
	6	0.60	0.60	0.54	0.75	0.65	0.63	0.73
	7	0.64	0.62	0.60	0.73	0.66	0.65	0.76
	8	0.69	0.66	0.64	0.70	0.62	0.65	0.76
	9	0.72	0.66	0.66	0.68	0.56	0.67	0.73
	10	0.76	0.70	0.68	0.66	0.48	0.69	0.73
	11	0.79	0.72	0.72	0.59	0.40	0.66	0.73
	12	0.76	0.74	0.74	0.62	0.30	0.66	0.71
	13	0.71	0.76	0.77	0.56	0.27	0.63	0.70
	14	0.71	0.79	0.80	0.55	0.27**	0.62	0.71
	15	0.69	0.76	0.78	0.55	0.25**	0.66	0.70
Tianjin City (phase 1, 2021. 12.26–02.20)	0	0.89	0.84	0.83	0.44	0.79	0.11*	0.46*
	1	0.84	0.81	0.82	0.47	0.80	0.09*	0.49
	2	0.77	0.77	0.81	0.44	0.72	0.13*	0.55
	3	0.70	0.73	0.78	0.46	0.64	0.19	0.65
	4	0.56	0.60	0.70	0.44	0.51	0.31**	0.66
	5	0.44	0.47	0.60	0.36**	0.46	0.33**	0.63
	6	0.34**	0.36**	0.50	0.31**	0.42	0.26**	0.63
	7	0.23*	0.31**	0.39	0.31**	0.36**	0.27**	0.63
	8	0.14*	0.24*	0.32**	0.27**	0.33**	0.19*	0.63
Tianjin City (phase 2, 2022. 02.11–04.26)	0	0.82	0.86	0.87	0.31**	0.65	0.24*	0.65
	1	0.72	0.79	0.84	0.23**	0.62	0.18*	0.59
	2	0.63	0.73	0.79	0.21*	0.52	0.15*	0.52
	3	0.55	0.65	0.74	0.16*	0.44	0.18*	0.46
	4	0.50	0.63	0.69	0.13*	0.42	0.09*	0.41
	5	0.43	0.60	0.62	0.18*	0.32	0.13*	0.33
	6	0.34	0.52	0.56	0.10*	0.27**	0.09*	0.26**
	7	0.26**	0.48	0.50	0.06*	0.22*	0.10*	0.21*
	8	0.20*	0.44	0.45	0.10*	0.20*	0.07*	0.20*
Shenzhen City (2022.01.27–04.30)	0	0.76	0.81	0.67	0.55	0.69	0.06*	0.37
	1	0.74	0.80	0.66	0.52	0.65	0.07*	0.35
	2	0.71	0.79	0.64	0.46	0.61	0.07*	0.33
	3	0.68	0.75	0.58	0.39	0.57	0.05*	0.26**
	4	0.61	0.70	0.53	0.35	0.52	0.05*	0.22**
	5	0.53	0.66	0.47	0.26	0.47	0.02*	0.19*
	6	0.43	0.61	0.40	0.17*	0.42	-0.01*	0.15*
	7	0.36	0.54	0.34	0.09*	0.36	-0.05*	0.09*
	8	0.29	0.49	0.26	0.04*	0.30	-0.10*	0.03*
Jilin Province (2022.02.16–04.30)	0	0.76	0.70	0.74	0.69	0.71	0.81	0.65
	1	0.80	0.75	0.79	0.74	0.75	0.80	0.70
	2	0.84	0.79	0.82	0.79	0.79	0.80	0.74
	3	0.87	0.83	0.85	0.84	0.82	0.77	0.76
	4	0.89	0.86	0.88	0.85	0.85	0.75	0.78
	5	0.90	0.88	0.89	0.84	0.88	0.74	0.79
	6	0.91	0.90	0.91	0.84	0.88	0.74	0.81
	7	0.91	0.91	0.91	0.84	0.88	0.67	0.81
	8	0.92	0.92	0.92	0.83	0.89	0.63	0.82
	9	0.90	0.91	0.91	0.81	0.86	0.60	0.79
	10	0.87	0.89	0.89	0.79	0.87	0.59	0.77
	11	0.85	0.87	0.86	0.75	0.83	0.55	0.72
	12	0.83	0.85	0.85	0.73	0.81	0.50	0.69
	13	0.80	0.82	0.82	0.70	0.79	0.46	0.66
	14	0.76	0.80	0.79	0.63	0.76	0.47	0.62
	15	0.73	0.78	0.77	0.61	0.72	0.46	0.60
Yanbian Prefecture (2022.02.16–04.06)	0	0.78	0.79	0.82	0.52	0.51	0.27*	0.64
	1	0.78	0.74	0.81	0.45	0.55	0.28*	0.58
	2	0.75	0.72	0.74	0.32**	0.56	0.30**	0.50
	3	0.63	0.61	0.67	0.35**	0.52	0.26*	0.55
	4	0.57	0.61	0.59	0.27*	0.45	0.21*	0.44
	5	0.51	0.51	0.54	0.19*	0.48	0.04*	0.40
	6	0.44	0.41	0.43	0.10*	0.46	-0.08*	0.39
	7	0.36**	0.35**	0.37**	0.01*	0.40	-0.07*	0.33**
	8	0.33**	0.32**	0.35**	-0.01*	0.22*	-0.04*	0.27*
Jilin Prefecture (2022.02.18–4.30)	0	0.86	0.66	0.81	0.58	0.71	0.63	0.69
	1	0.89	0.71	0.86	0.63	0.76	0.63	0.74
	2	0.91	0.77	0.89	0.67	0.80	0.58	0.77
	3	0.92	0.79	0.91	0.69	0.81	0.59	0.79
	4	0.93	0.80	0.92	0.66	0.79	0.52	0.78
	5	0.93	0.82	0.92	0.65	0.83	0.54	0.79
	6	0.92	0.81	0.91	0.62	0.79	0.51	0.82
	7	0.90	0.81	0.90	0.67	0.80	0.47	0.80
	8	0.89	0.79	0.88	0.64	0.78	0.40	0.80
	9	0.85	0.78	0.86	0.66	0.74	0.34	0.77
	10	0.81	0.74	0.82	0.61	0.72	0.33	0.73
	11	0.77	0.72	0.79	0.58	0.68	0.28**	0.70
	12	0.73	0.68	0.75	0.54	0.62	0.28**	0.66
	13	0.68	0.63	0.71	0.51	0.59	0.20*	0.61
	14	0.63	0.60	0.66	0.49	0.54	0.20*	0.57
	15	0.56	0.56	0.63	0.45	0.50	0.14*	0.51
Changchun Prefecture (2022.02.19–04.30)	0	0.63	0.54	0.56	0.54	0.56	0.73	0.62
	1	0.69	0.60	0.62	0.61	0.60	0.72	0.65
	2	0.74	0.66	0.68	0.65	0.67	0.72	0.67
	3	0.78	0.71	0.72	0.70	0.70	0.74	0.69
	4	0.81	0.75	0.77	0.77	0.75	0.78	0.71
	5	0.84	0.79	0.80	0.77	0.78	0.74	0.73
	6	0.87	0.82	0.84	0.76	0.81	0.76	0.74
	7	0.88	0.86	0.87	0.79	0.82	0.74	0.76
	8	0.90	0.88	0.88	0.79	0.84	0.71	0.77
	9	0.91	0.88	0.90	0.76	0.86	0.67	0.76
	10	0.90	0.89	0.90	0.76	0.87	0.65	0.73
	11	0.90	0.88	0.90	0.76	0.88	0.62	0.70
	12	0.87	0.88	0.90	0.73	0.86	0.57	0.67
	13	0.85	0.88	0.88	0.68	0.85	0.54	0.65
	14	0.82	0.86	0.87	0.65	0.84	0.56	0.62
	15	0.80	0.86	0.87	0.58	0.81	0.54	0.58
Shandong Province (2022.02.16–04.30)	0	0.84	0.83	0.85	0.76	0.76	0.44	0.79
	1	0.81	0.81	0.82	0.70	0.73	0.38	0.73
	2	0.78	0.78	0.78	0.63	0.66	0.35	0.68
	3	0.76	0.77	0.75	0.59	0.64	0.32**	0.64
	4	0.72	0.74	0.72	0.54	0.62	0.31**	0.60
	5	0.67	0.71	0.68	0.50	0.56	0.31**	0.58
	6	0.61	0.66	0.63	0.49	0.51	0.27**	0.54
	7	0.56	0.59	0.58	0.42	0.46	0.24**	0.49
	8	0.50	0.54	0.54	0.36	0.40	0.21*	0.41
Qingdao Prefecture (2022.02.16–04.30)	0	0.79	0.80	0.79	0.66	0.69	0.19	0.68
	1	0.75	0.73	0.76	0.68	0.63	0.10	0.61
	2	0.77	0.74	0.74	0.61	0.68	0.19*	0.57
	3	0.69	0.68	0.62	0.54	0.54	0.27**	0.48
	4	0.73	0.71	0.68	0.56	0.58	0.15*	0.50
	5	0.66	0.67	0.62	0.49	0.54	0.19*	0.45
	6	0.60	0.62	0.57	0.52	0.43	0.04*	0.41
	7	0.54	0.54	0.50	0.37	0.41	0.06*	0.33
	8	0.47	0.45	0.44	0.31**	0.33	0.001*	0.27**
Shanghai City (2022.02.16–04.30)	0	0.79	0.33	0.07*	0.79	0.39	0.86	0.20*
	1	0.81	0.38	0.12*	0.81	0.43	0.87	0.25**
	2	0.84	0.44	0.18*	0.82	0.48	0.88	0.30**
	3	0.86	0.49	0.23**	0.83	0.53	0.89	0.35
	4	0.87	0.53	0.29**	0.84	0.56	0.90	0.40
	5	0.88	0.57	0.33	0.85	0.59	0.90	0.44
	6	0.89	0.60	0.37	0.85	0.62	0.90	0.48
	7	0.89	0.64	0.42	0.86	0.63	0.91	0.52
	8	0.90	0.67	0.47	0.87	0.65	0.91	0.56
	9	0.89	0.70	0.52	0.88	0.68	0.92	0.60
	10	0.90	0.72	0.57	0.88	0.70	0.92	0.64
	11	0.89	0.73	0.60	0.88	0.72	0.92	0.67
	12	0.89	0.75	0.63	0.86	0.74	0.91	0.69
	13	0.88	0.76	0.67	0.85	0.77	0.91	0.73
	14	0.87	0.78	0.71	0.85	0.78	0.90	0.76
	15	0.85	0.78	0.73	0.84	0.79	0.89	0.78
	16	0.84	0.78	0.76	0.84	0.81	0.88	0.80
	17	0.84	0.79	0.78	0.84	0.81	0.87	0.82
	18	0.84	0.80	0.80	0.84	0.83	0.86	0.83
	19	0.82	0.81	0.81	0.82	0.82	0.84	0.83
	20	0.81	0.81	0.81	0.82	0.81	0.83	0.83
	21	0.79	0.81	0.82	0.82	0.80	0.82	0.82
	22	0.78	0.82	0.84	0.81	0.79	0.81	0.82
	23	0.77	0.83	0.84	0.82	0.77	0.79	0.82
	24	0.76	0.84	0.84	0.81	0.76	0.78	0.82
	25	0.75	0.84	0.85	0.82	0.75	0.78	0.81
	26	0.74	0.84	0.84	0.81	0.72	0.76	0.81
	27	0.72	0.83	0.83	0.80	0.72	0.75	0.79
	28	0.70	0.81	0.82	0.77	0.69	0.72	0.79
	29	0.67	0.78	0.79	0.75	0.65	0.68	0.77
	30	0.63	0.75	0.77	0.71	0.61	0.65	0.75
Fujian Province (2022.02.28–04.30)	0	0.88	0.79	0.81	0.93	0.91	0.68	0.88
	1	0.92	0.85	0.87	0.93	0.92	0.72	0.90
	2	0.93	0.88	0.91	0.89	0.92	0.76	0.91
	3	0.93	0.90	0.93	0.84	0.86	0.75	0.90
	4	0.89	0.89	0.92	0.79	0.81	0.71	0.86
	5	0.84	0.88	0.90	0.74	0.75	0.67	0.81
	6	0.77	0.85	0.86	0.68	0.69	0.58	0.76
	7	0.73	0.81	0.81	0.62	0.60	0.48	0.70
	8	0.67	0.77	0.76	0.55	0.50	0.42	0.65
Quanzhou Prefecture (2022.02.28–04.28)	0	0.91	0.91	0.90	0.61	0.88	0.59	0.90
	1	0.91	0.92	0.93	0.62	0.85	0.55	0.89
	2	0.90	0.92	0.92	0.61	0.84	0.53	0.90
	3	0.88	0.90	0.91	0.57	0.78	0.50	0.89
	4	0.83	0.87	0.88	0.50	0.72	0.40	0.84
	5	0.76	0.81	0.83	0.45	0.65	0.34**	0.78
	6	0.68	0.75	0.76	0.41	0.56	0.26**	0.72
	7	0.61	0.67	0.69	0.31**	0.45	0.21*	0.67
	8	0.53	0.59	0.62	0.22*	0.35**	0.15*	0.60

*P-value > 0.05; **P-value between 0.01 and 0.05; the less without mark with *P-value* < 0.001.

Compared to “Novel Coronavirus”, “COVID-19” and “COVID-19 epidemic”, weaker associations were found between the search volume of “Mask”, “Nucleic acid”, “COVID-19 vaccine” and “Asymptomatic patient” and the number of cases in the same study areas. The association in all the study areas (except Shanghai) were moderate (r_s_:0.40–0.69), while it was high association (r_s_:0.87–0.92) in Shanghai, . However, no association was found between the search volume of “COVID-19 vaccine” and the number of cases in Tianjin City (phase 1), Shenzhen City, Yanbian Korean Autonomous Prefecture, Shangdong Province and Qingdao Prefecture. And no association was found between the search volume of “mask” and the number of cases in Yanbian Korean Autonomous Prefecture and Tianjin City (phase 2).

### Lead time confirmed in negative binomial regression model

The search terms that had significant association with the number of cases were included in the factor analysis with different lead time. Only one factor was identified with cumulative contribution > 70% in all study areas. The variables with factor loadings > 0.5 were ranked by descending order and listed in Supplementary Table S1. A negative binomial regression with the factor scores as independent variable was conducted. The best models can be seen in 1 day lead time in Henan Province (phase 1), Tianjin City (phase 2) and Quanzhou Prefecture, 3 days lead time in Tianjin City (phase 1) and Fujian province, 5 days lead time in Jilin Prefecture, 6 days lead time in Jilin Province, 8 days lead time in Changchun Prefecture, 14 days lead time in Henan Province (phase 2), 26 days in Shanghai City and 0 day lead time in Shenzhen City, Shandong Province, Qingdao Prefecture and Yanbian Korean Autonomous Prefecture (Supplementary Table S1).

## Discussion

This study was conducted on 1 January to 30 April, 2022, from the beginning of the Omicron variant being detected until it dominated the COVID-19 epidemic globally. And it was also in the period that massive nucleic acid testing action was regular implemented in China. With the variant strain changed and the great improvement in the timeliness of diagnosis. We explored the lead time and indicators of Baidu Search Index based on several severe epidemics in China. Our study found that the COVID-19 epidemic can be early warning through the Baidu search behavior, but the lead time had spatial and temporal variation. With the implementation of massive nucleic acid testing, the pre-warning time was shortened to 0–3 days if without medical resource shortage. While the earlier effect could be 28 days when suffering extreme shortage of medical resource. This finding may contribute to the design of early warning system and implicate the need of strengthen the disease surveillance when perceiving Baidu Search Index increase.

On January, the Baidu search volume of “COVID-19 epidemic” and “Novel Coronavirus” showed 3 days earlier than the epidemic in Henan Province (phase 1) and Tianjin City (phase 1). The lead time was shorter than previous study in China and other countries^11,12,15,16^. This may due to the previous studies were conducted in the first half year of 2020 with limited knowledge about this emergence infectious disease and without massive nucleic acid testing action. The timeliness of diagnosis was obviously delayed in that period. The abnormally high search volume of “Novel Coronavirus” and “COVID-19 epidemic” in Tianjin and Shenzhen City, which was as high as 889–1793 per 1,000,000 population, may due to the local Omicron infection cases in China was firstly detected in Tianjin City on January 8^27^ and in Shenzhen on 16 January^31^. People in these two areas pay great attention to the new variants strain and the epidemic situation. By contrast, although the number of cases in Henan Province was twice higher than that of Tianjin City, but the search volume was less than one fifth that in Tianjin City. This may due to Tianjin City is the municipalities directly under the Central Government, the economic level and the proportion of young people in Tianjin City is higher than Henan Province. Moreover, the epidemic in Henan Province was caused by Delta variant, which has become a dominant around the world since mid-2021^32^. People may pay less attention to this familiar strain. Thus, the search peak of “COVID-19 epidemic” in Henan Province was higher than “Novel Coronavirus”. While in the other areas without cases reported, a smaller search peak can be observed. It was suddenly increased on 9 January and peak on 10–12 January. This may be affected by the announcement of detection of local Omicron cases in China on 8 January^27^. The public was curious about the new variant strain and tended to search for related information even did not have associated symptoms^33^, so the search peak of “Novel Coronavirus” was higher than “COVID-19 epidemic” in these areas.

The lead time of Baidu Search Index had spatial and temporal variation. The lead time in Shanghai City was 4 weeks and in Henan province (second phrase) was 2 weeks while in Jilin Province, Jilin Prefecture and Changchun Prefecture was 5–8 days. The variation may be close related to the medical resource, the timeliness of diagnosis and public sensitivities. Shanghai was the most serious epidemic areas in China in the study period, which experienced extreme shortage of testing capacity in the beginning of epidemic. With more than 38,000 medical workers across the country being sent to Shanghai in early April^34^, over 550,000 cases were detected, but the timeless of diagnosis was obviously delayed. Thus, the lead time in Shanghai was obviously longer than the other areas, a high association of Baidu search volume and the number of cases can be observed in 14–28 days lead time in Shanghai. Although the epidemic was also challenging in Jilin Province (77,080 cases), but which still not leading to serious medical resources shortage, the lead time was much shorter than Shanghai City. However, the more serious epidemic area in Jilin Province, the longer lead time and higher search volume can be observed (Changchun Prefecture > Jilin Prefecture > Jilin Province). It should be noted that although only dozens of cases in the second epidemic peak of Henan Province (phrase 2), but a long lead time can be observed. This may be due to a local epidemic in Henan Province (phrase 1) in January, which increased the public’s sensitivity and advanced the search behavior once any case was reported. In Henan Province (phase 1), Tianjin City (phase 1& phase 2), Quanzhou Prefecture and Fujian Province, where the epidemic was relatively mild and the medical resource was adequate, only 1–3 days lead time can be observed. This was obviously shorter than the previous studies^11,12,15–17,35,36^, which may due to the regular implement of massive nucleic acid testing improve the timeliness of cases detection. This phenomenon was more prominent in Shandong Province and Qingdao Prefecture, where majority reported cases were asymptomatic. This implicated that mostly cases were being detected from nucleic acid screening. Thus, 0 day lead time can be observed. Moreover, the lead time was also 0 day in Shenzhen City and Yanbian Korean Autonomous Prefecture. This may because Shenzhen is the most open special economic zone in China. It was the first city in China launch to build a “15- minute walk nucleic acid service circle” on 11 March 2022, which enable all citizens in any part of the city to access a testing site within a 15-min walk. And three rounds of full citizens nucleic acid testing were conducted on 14–20 March^37^. While Yanbian Korean Autonomous Prefecture is located in Northeast China with 11 ports to North Korea and Russia and the only water channel from inland China to the Sea of Japan. Strong control measures were taken in the border areas and the massive nucleic acid testing in Shenzhen City may largely improve the timeliness of cases detection.

Similar to the previous findings, the strength of correlation was different for different keywords^12^. The Baidu search volume of “COVID-19 epidemic”, “Novel Coronavirus” and “COVID-19” was highly correlated (r_s_:0.70–0.93) with the number of cases during the epidemic period concerned with different lead times, while the search terms of “Mask”, “Nucleic acid”, “COVID-19 vaccine” and “Asymptomatic patient” showed a weaker association in the same study areas. An explanation could be that public pay less attention to the familiar control strategies, such as wearing mask, test nucleic acid, and injection COVID-19 vaccine and not much concerned about the asymptomatic infection. Moreover, the strength of correlation was changed over time. The highest association between the search volume and the number of cases was consistent with the lead time that being observed from the epidemic curve. The same pattern of the changed in epidemiological trend implicated that Baidu Search Index could be used in monitoring the epidemic trend. This was further confirmed as the best model fit of Baidu Search Index and the number of cases in each study area was also showed in the observed lead time.

### Limitations

This across a wide range of geographical areas study showed that Baidu Search Index can be used in the early warning of COVID-19 epidemic. However, the indicators and lead time had spatial and temporal variation. Thus, the indicators and the pre-warming time being detected in this study may not suitable to the other areas or other study periods on account of the sensitivity of surveillance systems, medical resource allocation, nucleic acid testing actions, specific control measures and other confounders. Another limitation is that we used cases data public by the health department, the accuracy and the timeliness of reported cases was unevaluated, which may be affected by the tricky situation of local epidemic. However, the whole epidemic trend in a certain area would not change by the slightly underreported. And considering the geographical variation of the timeliness of cases reporting, we explored the lead time in different areas.

## Conclusion

The Baidu Search Index increased ahead of the number of cases implicated that it could be used in the early warning of COVID-19. The lead time and indicators of Baidu Search index had spatial and temporal variation, which change with the variants strain, nucleic acid testing actions, medical resource allocation, and other control policy. We should focus on multiple indicators when design the early warning system and strengthen the disease surveillance when perceiving the increase of internet search volume, especially for the emerging and re-emerging infectious diseases.

### Supplementary Information

Supplementary Table S1.

## Data Availability

Please contact corresponding author for data requests.
