# Author Correction: The lead time and geographical variations of Baidu Search Index in the early warning of COVID-19

**DOI:** 10.1038/s41598-023-43165-z

**Published:** 2023-09-28

**Authors:** Yuhua Ruan, Tengda Huang, Wanwan Zhou, Jinhui Zhu, Qiuyu Liang, Lixian Zhong, Xiaofen Tang, Lu Liu, Shiwen Chen, Yihong Xie

**Affiliations:** 1 State Key Laboratory of Infectious Disease Prevention and Control (SKLID), National Center for AIDS/STD Control and Prevention (NCAIDS), Chinese Center for Disease Control and Prevention (China CDC), Collaborative Innovation Center for Diagnosis and Treatment of Infectious Diseases, Beijing, China; 2 Department of Epidemiology and Biostatistics, Guangxi Medical University, Nanning, China; 3 Guangxi Key Laboratory of Major Infectious Disease Prevention Control and Biosafety Emergency Response, Guangxi Center for Disease Control and Prevention, Nanning, China; 4 Department of Health Management, The People’s Hospital of Guangxi Zhuang Autonomous Region & Research Center of Health Management, Guangxi Academy of Medical Sciences, Nanning, China; 5 Guangxi Colleges and Universities Key Laboratory of Prevention and Control of Highly Prevalent Diseases, Guangxi Medical University, Nanning, China

Correction to: *Scientific Reports* 10.1038/s41598-023-41939-z, published online 07 September 2023

The Acknowledgements section in the original version of this Article was omitted. The Acknowledgements section now reads:

“This work was supported by Health and Emergency Skills Training Center of Guangxi (HESTCG202104), National Natural Science Foundation of China [11971479], Guangxi Bagui Honor Scholarship, and Chinese State Key Laboratory of Infectious Disease Prevention and Control. The funders had no role in study design, data collection and analysis, decision to publish, or preparation of the manuscript.”

The original Article has been corrected.

