# Supplementary material for: The lead time and geographical variations of Baidu Search Index in the early warning of COVID-19
Source: Sci Rep. 2023 Sep 7;13:14705. doi: 10.1038/s41598-023-41939-z (PMC10484897; doi:10.1038/s41598-023-41939-z)
Supplement: Supplementary file 1 — Supplementary Table S1. [file 41598_2023_41939_MOESM1_ESM.docx]

Supplementary Table S1. The factor analysis and negative binomial regression model result in each study areas with different lead time

| Study area | Lead time (day) | Variables with factor loading >0.5 | Cumulative Proportion for factor analysis | AIC value for negative binomial regression model |
| --- | --- | --- | --- | --- |
| Henan Province  (phase 1) | 0 | COVID-19 epidemic, COVID-19, Nucleic Acid, Mask, Asymptomatic Patient, Novel Coronavirus | 0.724 | 42.09 |
|  | 1 | COVID-19 epidemic, COVID-19, Nucleic Acid, Mask, Asymptomatic Patient, Novel Coronavirus | 0.726 | 41.80 |
|  | 2 | COVID-19 epidemic, COVID-19, Nucleic Acid, Mask, Asymptomatic Patient, Novel Coronavirus | 0.725 | 42.14 |
|  | 3 | COVID-19 epidemic, COVID-19, Nucleic Acid, Mask, Asymptomatic Patient, Novel Coronavirus | 0.723 | 43.64 |
|  | 4 | COVID-19 epidemic, COVID-19, Nucleic Acid, Mask, Asymptomatic Patient, Novel Coronavirus | 0.720 | 45.35 |
|  | 5 | COVID-19 epidemic,COVID-19, Nucleic Acid, Mask, Asymptomatic Patient, Novel Coronavirus | 0.720 | 47.41 |
|  | 6 | COVID-19 epidemic, COVID-19, Nucleic Acid, Mask, Asymptomatic Patient, Novel Coronavirus | 0.719 | 50.31 |
|  | 7 | COVID-19 epidemic, COVID-19, Nucleic Acid, Mask, Asymptomatic Patient, Novel Coronavirus | 0.719 | 53.06 |
|  | 8 | COVID-19 epidemic, COVID-19, Nucleic Acid, Mask, Asymptomatic Patient, Novel Coronavirus | 0.717 | 55.86 |
| Henan Province  (phase 2) | 0 | Novel Coronavirus, COVID-19, COVID-19 epidemic, Nucleic Acid, Mask, Asymptomatic Patient, vaccine | 0.709 | 49.92 |
|  | 1 | Novel Coronavirus, COVID-19, COVID-19 epidemic, Nucleic Acid, Mask, Asymptomatic Patient, vaccine | 0.712 | 49.65 |
|  | 2 | Novel Coronavirus, COVID-19, COVID-19 epidemic, Nucleic Acid, Mask, Asymptomatic Patient, vaccine | 0.717 | 49.23 |
|  | 3 | Novel Coronavirus, COVID-19, COVID-19 epidemic, Nucleic Acid, Mask, Asymptomatic Patient, vaccine | 0.720 | 49.05 |
|  | 4 | Novel Coronavirus, COVID-19, COVID-19 epidemic, Nucleic Acid, Mask, Asymptomatic Patient, vaccine | 0.725 | 48.89 |
|  | 5 | Novel Coronavirus, COVID-19, COVID-19 epidemic, Mask, Nucleic Acid, Asymptomatic Patient, vaccine | 0.727 | 48.25 |
|  | 6 | Novel Coronavirus, COVID-19, COVID-19 epidemic, Mask, Asymptomatic Patient, Nucleic Acid, vaccine | 0.728 | 48.04 |
|  | 7 | Novel Coronavirus, COVID-19, COVID-19 epidemic, Mask, Asymptomatic Patient, Nucleic Acid, vaccine | 0.729 | 47.52 |
|  | 8 | Novel Coronavirus, COVID-19, COVID-19 epidemic, Mask, Asymptomatic Patient, Nucleic Acid, vaccine | 0.725 | 47.05 |
|  | 9 | Novel Coronavirus, COVID-19, COVID-19 epidemic, Mask, Asymptomatic Patient, vaccine, Nucleic Acid | 0.720 | 46.57 |
|  | 10 | COVID-19, COVID-19 epidemic, Novel Coronavirus, Mask, Asymptomatic Patient, vaccine, Nucleic Acid | 0.719 | 46.89 |
|  | 11 | COVID-19, COVID-19 epidemic, Novel Coronavirus, Mask, Asymptomatic Patient, vaccine | 0.721 | 46.54 |
|  | 12 | COVID-19, COVID-19 epidemic, Novel Coronavirus, Mask, Asymptomatic Patient, vaccine | 0.727 | 46.21 |
|  | 13 | COVID-19, COVID-19 epidemic, Novel Coronavirus, Mask, Asymptomatic Patient, vaccine | 0.733 | 45.87 |
|  | 14 | COVID-19, COVID-19 epidemic, Novel Coronavirus, Mask, Asymptomatic Patient, vaccine | 0.737 | 45.51 |
|  | 15 | COVID-19, COVID-19 epidemic,, Novel Coronavirus, Mask, Asymptomatic Patient, vaccine, Nucleic Acid | 0.742 | 45.91 |
| Tianjin (phase 1) | 0 | COVID-19 epidemic, COVID-19, Nucleic Acid, Novel Coronavirus, Asymptomatic Patient, Mask | 0.814 | 99.04 |
|  | 1 | COVID-19 epidemic, COVID-19, Nucleic Acid, Novel Coronavirus, Asymptomatic Patient, Mask | 0.812 | 94.716 |
|  | 2 | COVID-19 epidemic, COVID-19, Nucleic Acid, Novel Coronavirus, Asymptomatic Patient, Mask | 0.814 | 93.83 |
|  | 3 | COVID-19 epidemic, COVID-19, Nucleic Acid, Novel Coronavirus, Asymptomatic Patient, Mask | 0.815 | 90.48 |
|  | 4 | COVID-19 epidemic, COVID-19, Nucleic Acid, Novel Coronavirus, Asymptomatic Patient, Mask | 0.814 | 100.00 |
|  | 5 | COVID-19 epidemic, COVID-19, Nucleic Acid, Novel Coronavirus, Asymptomatic Patient, Mask | 0.812 | 102.87 |
|  | 6 | COVID-19 epidemic, COVID-19, Nucleic Acid, Novel Coronavirus, Asymptomatic Patient, Mask | 0.814 | 110.81 |
|  | 7 | COVID-19 epidemic, COVID-19, Nucleic Acid, Novel Coronavirus, Asymptomatic Patient, Mask | 0.816 | 115.66 |
|  | 8 | COVID-19 epidemic, COVID-19, Nucleic Acid, Novel Coronavirus, Asymptomatic Patient, Mask | 0.817 | 121.39 |
| Tianjin (phase 2) | 0 | Novel Coronavirus, COVID-19 epidemic, COVID-19, Nucleic Acid, Asymptomatic Patient | 0.832 | 132.43 |
|  | 1 | Novel Coronavirus, COVID-19 epidemic, COVID-19, Nucleic Acid, Asymptomatic Patient | 0.832 | 133.67 |
|  | 2 | Novel Coronavirus, COVID-19 epidemic, COVID-19, Nucleic Acid, Asymptomatic Patient | 0.832 | 144.50 |
|  | 3 | Novel Coronavirus, COVID-19 epidemic, COVID-19, Nucleic Acid, Asymptomatic Patient | 0.833 | 157.24 |
|  | 4 | Novel Coronavirus, COVID-19 epidemic, COVID-19, Nucleic Acid, Asymptomatic Patient | 0.834 | 169.76 |
|  | 5 | Novel Coronavirus, COVID-19 epidemic, COVID-19, Nucleic Acid, Asymptomatic Patient | 0.835 | 179.04 |
|  | 6 | Novel Coronavirus, COVID-19 epidemic, COVID-19, Nucleic Acid, Asymptomatic Patient | 0.833 | 186.80 |
|  | 7 | Novel Coronavirus, COVID-19, COVID-19 epidemic, Nucleic Acid, Asymptomatic Patient | 0.829 | 190.93 |
|  | 8 | COVID-19, Novel Coronavirus, COVID-19 epidemic, Nucleic Acid, Asymptomatic Patient | 0.824 | 193.11 |
| Shenzhen City | 0 | COVID-19, Novel Coronavirus, COVID-19 epidemic, Nucleic Acid, Mask | 0.855 | 148.66 |
|  | 1 | COVID-19, Novel Coronavirus, COVID-19 epidemic, Nucleic Acid, Mask | 0.855 | 152.11 |
|  | 2 | COVID-19, Novel Coronavirus, COVID-19 epidemic, Nucleic Acid, Mask | 0.855 | 159.08 |
|  | 3 | COVID-19, Novel Coronavirus, COVID-19 epidemic, Nucleic Acid, Mask | 0.855 | 175.79 |
|  | 4 | COVID-19, COVID-19 epidemic, Novel Coronavirus, Nucleic Acid, Mask | 0.854 | 192.98 |
|  | 5 | COVID-19, COVID-19 epidemic, Novel Coronavirus, Nucleic Acid, Mask | 0.852 | 206.20 |
|  | 6 | COVID-19, COVID-19 epidemic, Novel Coronavirus, Nucleic Acid, Mask | 0.842 | 216.86 |
|  | 7 | COVID-19 epidemic, COVID-19, Nucleic Acid, Mask | 0.767 | 225.17 |
|  | 8 | COVID-19, COVID-19 epidemic, Novel Coronavirus, Nucleic Acid, Mask | 0.746 | 229.34 |
| Jilin Province | 0 | COVID-19 epidemic, Novel Coronavirus, COVID-19, Nucleic Acid, Asymptomatic Patient, Mask, vaccine | 0.790 | 638.60 |
|  | 1 | COVID-19 epidemic, Novel Coronavirus, COVID-19, Nucleic Acid, Asymptomatic Patient, Mask, vaccine | 0.802 | 629.95 |
|  | 2 | COVID-19 epidemic, Novel Coronavirus, COVID-19, Nucleic Acid, Asymptomatic Patient, Mask, vaccine | 0.809 | 620.91 |
|  | 3 | COVID-19 epidemic, Novel Coronavirus, COVID-19, Nucleic Acid, Asymptomatic Patient, Mask, vaccine | 0.820 | 610.07 |
|  | 4 | COVID-19 epidemic, Novel Coronavirus, COVID-19, Nucleic Acid, Asymptomatic Patient, Mask, vaccine | 0.826 | 600.40 |
|  | 5 | Novel Coronavirus, COVID-19 epidemic, COVID-19, Nucleic Acid, Asymptomatic Patient, Mask, vaccine | 0.830 | 592.17 |
|  | 6 | Novel Coronavirus, COVID-19 epidemic, COVID-19, Nucleic Acid, Asymptomatic Patient, Mask, vaccine | 0.832 | 585.13 |
|  | 7 | Novel Coronavirus, COVID-19 epidemic, COVID-19, Nucleic Acid, Asymptomatic Patient, Mask, vaccine | 0.834 | 586.91 |
|  | 8 | Novel Coronavirus, COVID-19 epidemic, COVID-19, Nucleic Acid, Asymptomatic Patient, Mask, vaccine | 0.838 | 586.47 |
|  | 9 | Novel Coronavirus, COVID-19 epidemic, COVID-19, Nucleic Acid, Asymptomatic Patient, Mask, vaccine | 0.841 | 600.99 |
|  | 10 | Novel Coronavirus, COVID-19 epidemic, COVID-19, Nucleic Acid, Asymptomatic Patient, Mask, vaccine | 0.844 | 620.70 |
|  | 11 | Novel Coronavirus, COVID-19 epidemic, COVID-19, Nucleic Acid, Asymptomatic Patient, Mask, vaccine | 0.847 | 632.95 |
|  | 12 | Novel Coronavirus, COVID-19 epidemic, COVID-19, Nucleic Acid, Mask, Asymptomatic Patient, vaccine | 0.851 | 647.00 |
|  | 13 | Novel Coronavirus, COVID-19 epidemic, COVID-19, Nucleic Acid, Mask, Asymptomatic Patient, vaccine | 0.855 | 652.14 |
|  | 14 | Novel Coronavirus, COVID-19 epidemic, COVID-19, Nucleic Acid, Mask, Asymptomatic Patient, vaccine | 0.858 | 657.72 |
|  | 15 | Novel Coronavirus, COVID-19 epidemic, COVID-19, Nucleic Acid, Mask, Asymptomatic Patient, vaccine | 0.861 | 661.10 |
| Yanbian Prefecture of Jilin Province | 0 | COVID-19 epidemic, Novel Coronavirus, COVID-19, Asymptomatic Patient, Nucleic Acid | 0.755 | 152.14 |
|  | 1 | COVID-19 epidemic, Novel Coronavirus, COVID-19, Asymptomatic Patient, Nucleic Acid | 0.772 | 158.94 |
|  | 2 | COVID-19 epidemic, Novel Coronavirus, COVID-19, Asymptomatic Patient, Nucleic Acid | 0.784 | 169.49 |
|  | 3 | COVID-19 epidemic, Novel Coronavirus, COVID-19, Asymptomatic Patient, Nucleic Acid | 0.785 | 178.63 |
|  | 4 | COVID-19 epidemic, Novel Coronavirus, COVID-19, Asymptomatic Patient, Nucleic Acid | 0.791 | 183.55 |
|  | 5 | COVID-19 epidemic, Novel Coronavirus, COVID-19, Asymptomatic Patient, Nucleic Acid | 0.794 | 186.81 |
|  | 6 | COVID-19 epidemic, Novel Coronavirus, COVID-19, Asymptomatic Patient, Nucleic Acid | 0.801 | 189.43 |
|  | 7 | COVID-19 epidemic, Novel Coronavirus, COVID-19, Asymptomatic Patient, Nucleic Acid | 0.806 | 190.46 |
|  | 8 | COVID-19 epidemic, Novel Coronavirus, Asymptomatic Patient, COVID-19, Nucleic Acid | 0.801 | 190.86 |
| Jilin Prefecture of Jilin Province | 0 | COVID-19 epidemic, Novel Coronavirus, COVID-19, Asymptomatic Patient, Nucleic Acid, Mask | 0.699 | 671.62 |
|  | 1 | COVID-19 epidemic, Novel Coronavirus, COVID-19, Asymptomatic Patient, Nucleic Acid, Mask | 0.706 | 661.09 |
|  | 2 | COVID-19 epidemic, Novel Coronavirus, COVID-19, Asymptomatic Patient, Nucleic Acid, Mask | 0.717 | 654.67 |
|  | 3 | COVID-19 epidemic, Novel Coronavirus, COVID-19, Asymptomatic Patient, Nucleic Acid, Mask | 0.721 | 646.90 |
|  | 4 | COVID-19 epidemic, Novel Coronavirus, COVID-19, Asymptomatic Patient, Nucleic Acid, Mask | 0.722 | 643.64 |
|  | 5 | COVID-19 epidemic, Novel Coronavirus, COVID-19, Asymptomatic Patient, Nucleic Acid, Mask | 0.725 | 641.60 |
|  | 6 | COVID-19 epidemic, Novel Coronavirus, COVID-19, Asymptomatic Patient, Nucleic Acid, Mask | 0.729 | 645.08 |
|  | 7 | COVID-19 epidemic, Novel Coronavirus, COVID-19, Asymptomatic Patient, Nucleic Acid, Mask | 0.736 | 654.85 |
|  | 8 | COVID-19 epidemic, Novel Coronavirus, COVID-19, Asymptomatic Patient, Nucleic Acid, Mask | 0.738 | 663.80 |
|  | 9 | COVID-19 epidemic, Novel Coronavirus, COVID-19, Asymptomatic Patient, Nucleic Acid, Mask | 0.740 | 673.54 |
|  | 10 | COVID-19 epidemic, Novel Coronavirus, COVID-19, Asymptomatic Patient, Nucleic Acid, Mask | 0.745 | 687.77 |
|  | 11 | COVID-19 epidemic, Novel Coronavirus, COVID-19, Asymptomatic Patient, Nucleic Acid, Mask | 0.750 | 698.38 |
|  | 12 | COVID-19 epidemic, Novel Coronavirus, COVID-19, Asymptomatic Patient, Nucleic Acid, Mask | 0.754 | 709.04 |
|  | 13 | COVID-19 epidemic, Novel Coronavirus, COVID-19, Asymptomatic Patient, Nucleic Acid, Mask | 0.758 | 713.97 |
|  | 14 | COVID-19 epidemic, Novel Coronavirus, COVID-19, Asymptomatic Patient, Nucleic Acid, Mask | 0.764 | 717.19 |
|  | 15 | COVID-19 epidemic, Novel Coronavirus, COVID-19, Asymptomatic Patient, Nucleic Acid, Mask | 0.767 | 719.10 |
| Changchun Prefecture of Jilin Province | 0 | COVID-19 epidemic, COVID-19, Novel Coronavirus, Nucleic Acid, Asymptomatic Patient, vaccine, Mask | 0.737 | 702.65 |
|  | 1 | COVID-19 epidemic, COVID-19, Novel Coronavirus, Nucleic Acid, Asymptomatic Patient, vaccine, Mask | 0.747 | 698.22 |
|  | 2 | COVID-19 epidemic, COVID-19, Novel Coronavirus, Nucleic Acid, Asymptomatic Patient, vaccine, Mask | 0.759 | 692.65 |
|  | 3 | COVID-19 epidemic, COVID-19, Novel Coronavirus, Nucleic Acid, Asymptomatic Patient, Mask, vaccine | 0.773 | 685.52 |
|  | 4 | COVID-19 epidemic, COVID-19, Novel Coronavirus, Nucleic Acid, Asymptomatic Patient, Mask, vaccine | 0.783 | 677.30 |
|  | 5 | COVID-19 epidemic, Novel Coronavirus, COVID-19, Nucleic Acid, Asymptomatic Patient, Mask, vaccine | 0.788 | 668.89 |
|  | 6 | COVID-19 epidemic, Novel Coronavirus, COVID-19, Nucleic Acid, Asymptomatic Patient, Mask, vaccine | 0.795 | 658.17 |
|  | 7 | COVID-19 epidemic, Novel Coronavirus, COVID-19, Nucleic Acid, Asymptomatic Patient, Mask, vaccine | 0.800 | 649.9 |
|  | 8 | COVID-19 epidemic, Novel Coronavirus, COVID-19, Nucleic Acid, Mask, Asymptomatic Patient, vaccine | 0.804 | 641.44 |
|  | 9 | COVID-19 epidemic, Novel Coronavirus, COVID-19, Nucleic Acid, Mask, Asymptomatic Patient, vaccine | 0.809 | 646.48 |
|  | 10 | COVID-19 epidemic, Novel Coronavirus, COVID-19, Nucleic Acid, Mask, Asymptomatic Patient, vaccine | 0.813 | 658.96 |
|  | 11 | COVID-19 epidemic, Novel Coronavirus, COVID-19, Nucleic Acid, Mask, Asymptomatic Patient, vaccine | 0.817 | 665.27 |
|  | 12 | COVID-19 epidemic, Novel Coronavirus, COVID-19, Nucleic Acid, Mask, Asymptomatic Patient, vaccine | 0.823 | 675.06 |
|  | 13 | COVID-19 epidemic, Novel Coronavirus, COVID-19, Nucleic Acid, Mask, Asymptomatic Patient, vaccine | 0.828 | 681.16 |
|  | 14 | COVID-19 epidemic, Novel Coronavirus, COVID-19, Nucleic Acid, Mask, Asymptomatic Patient, vaccine | 0.832 | 683.29 |
|  | 15 | COVID-19 epidemic, Novel Coronavirus, COVID-19, Nucleic Acid, Mask, Asymptomatic Patient, vaccine | 0.836 | 687.10 |
| Shandong Province | 0 | COVID-19 epidemic, COVID-19, Novel Coronavirus, Mask, Asymptomatic Patient, Nucleic Acid | 0.897 | 139.75 |
|  | 1 | COVID-19 epidemic, COVID-19, Novel Coronavirus, Mask, Asymptomatic Patient, Nucleic Acid | 0.899 | 146.64 |
|  | 2 | COVID-19 epidemic, COVID-19, Novel Coronavirus, Mask, Asymptomatic Patient, Nucleic Acid | 0.901 | 153.48 |
|  | 3 | COVID-19 epidemic, COVID-19, Novel Coronavirus, Mask, Asymptomatic Patient, Nucleic Acid | 0.904 | 156.80 |
|  | 4 | COVID-19 epidemic, COVID-19, Novel Coronavirus, Mask, Asymptomatic Patient, Nucleic Acid | 0.908 | 157.10 |
|  | 5 | COVID-19 epidemic, COVID-19, Novel Coronavirus, Mask, Asymptomatic Patient, Nucleic Acid | 0.911 | 160.03 |
|  | 6 | COVID-19 epidemic, COVID-19, Novel Coronavirus, Mask, Asymptomatic Patient, Nucleic Acid | 0.913 | 166.64 |
|  | 7 | COVID-19 epidemic, COVID-19, Novel Coronavirus, Mask, Asymptomatic Patient, Nucleic Acid | 0.916 | 173.14 |
|  | 8 | COVID-19 epidemic, COVID-19, Novel Coronavirus, Mask, Asymptomatic Patient, Nucleic Acid | 0.919 | 176.67 |
| Qingdao Prefecture of Shandong Province | 0 | COVID-19 epidemic, COVID-19, Novel Coronavirus, Nucleic Acid, Asymptomatic Patient, Mask | 0.858 | 156.06 |
|  | 1 | COVID-19 epidemic, COVID-19, Novel Coronavirus, Nucleic Acid, Asymptomatic Patient, Mask | 0.859 | 192.01 |
|  | 2 | COVID-19 epidemic, COVID-19, Novel Coronavirus, Nucleic Acid, Asymptomatic Patient, Mask | 0.859 | 218.10 |
|  | 3 | COVID-19 epidemic, COVID-19, Novel Coronavirus, Nucleic Acid, Asymptomatic Patient, Mask | 0.862 | 227.61 |
|  | 4 | COVID-19 epidemic, COVID-19, Novel Coronavirus, Nucleic Acid, Asymptomatic Patient, Mask | 0.864 | 234.38 |
|  | 5 | COVID-19 epidemic, COVID-19, Novel Coronavirus, Nucleic Acid, Asymptomatic Patient, Mask | 0.866 | 243.05 |
|  | 6 | COVID-19 epidemic, COVID-19, Novel Coronavirus, Nucleic Acid, Asymptomatic Patient, Mask | 0.869 | 252.07 |
|  | 7 | COVID-19 epidemic, COVID-19, Novel Coronavirus, Nucleic Acid, Asymptomatic Patient, Mask | 0.870 | 256.52 |
|  | 8 | COVID-19 epidemic, COVID-19, Novel Coronavirus, Nucleic Acid, Asymptomatic Patient, Mask | 0.873 | 256.82 |
| Shanghai City | 0 | COVID-19, Nucleic Acid, COVID-19 epidemic, Asymptomatic Patient, Novel Coronavirus, Mask | 0.711 | 36971.00 |
|  | 1 | COVID-19, Nucleic Acid, COVID-19 epidemic, Asymptomatic Patient, Novel Coronavirus, Mask | 0.715 | 36444.00 |
|  | 2 | COVID-19, Nucleic Acid, COVID-19 epidemic, Asymptomatic Patient, Novel Coronavirus, Mask | 0.720 | 35704.00 |
|  | 3 | COVID-19, Nucleic Acid, COVID-19 epidemic, Asymptomatic Patient, Novel Coronavirus, Mask | 0.731 | 34799.00 |
|  | 4 | COVID-19, Nucleic Acid, COVID-19 epidemic, Asymptomatic Patient, Mask, Novel Coronavirus | 0.740 | 33709.00 |
|  | 5 | COVID-19, Nucleic Acid, COVID-19 epidemic, Asymptomatic Patient, Mask, Novel Coronavirus | 0.749 | 32591.00 |
|  | 6 | COVID-19, Nucleic Acid, COVID-19 epidemic, Asymptomatic Patient, Mask, Novel Coronavirus | 0.756 | 31524.00 |
|  | 7 | COVID-19, Nucleic Acid, COVID-19 epidemic, Asymptomatic Patient, Mask, Novel Coronavirus | 0.762 | 30235.00 |
|  | 8 | COVID-19, Nucleic Acid, COVID-19 epidemic, Asymptomatic Patient, Mask, Novel Coronavirus | 0.768 | 28690.00 |
|  | 9 | COVID-19, Nucleic Acid, COVID-19 epidemic, Asymptomatic Patient, Mask, Novel Coronavirus | 0.775 | 27279.00 |
|  | 10 | COVID-19, Nucleic Acid, COVID-19 epidemic, Asymptomatic Patient, Mask, Novel Coronavirus | 0.782 | 25849.00 |
|  | 11 | COVID-19, Nucleic Acid, COVID-19 epidemic, Asymptomatic Patient, Mask, Novel Coronavirus, vaccine | 0.788 | 24534.00 |
|  | 12 | COVID-19, Nucleic Acid, COVID-19 epidemic, Asymptomatic Patient, Mask, Novel Coronavirus, vaccine | 0.794 | 23352.00 |
|  | 13 | COVID-19, Nucleic Acid, COVID-19 epidemic, Asymptomatic Patient, Mask, Novel Coronavirus, vaccine | 0.801 | 22131.00 |
|  | 14 | COVID-19, Nucleic Acid, COVID-19 epidemic, Asymptomatic Patient, Mask, Novel Coronavirus, vaccine | 0.807 | 20525.00 |
|  | 15 | COVID-19, Nucleic Acid, COVID-19 epidemic, Asymptomatic Patient, Mask, Novel Coronavirus, vaccine | 0.812 | 19516.00 |
|  | 16 | COVID-19, Nucleic Acid, COVID-19 epidemic, Mask, Asymptomatic Patient, Novel Coronavirus, vaccine | 0.819 | 18704.00 |
|  | 17 | COVID-19, Nucleic Acid, COVID-19 epidemic, Mask, Asymptomatic Patient, Novel Coronavirus, vaccine | 0.825 | 17649.00 |
|  | 18 | COVID-19, Nucleic Acid, COVID-19 epidemic, Mask, Asymptomatic Patient, Novel Coronavirus, vaccine | 0.829 | 16500.00 |
|  | 19 | COVID-19, COVID-19 epidemic, Nucleic Acid, Mask, Asymptomatic Patient, Novel Coronavirus, vaccine | 0.833 | 15454.00 |
|  | 20 | COVID-19, COVID-19 epidemic, Mask, Nucleic Acid, Asymptomatic Patient, Novel Coronavirus, vaccine | 0.837 | 14381.00 |
|  | 21 | COVID-19, COVID-19 epidemic, Mask, Nucleic Acid, Asymptomatic Patient, Novel Coronavirus, vaccine | 0.839 | 13315.00 |
|  | 22 | COVID-19, COVID-19 epidemic, Mask, Nucleic Acid, Asymptomatic Patient, Novel Coronavirus, vaccine | 0.840 | 11863.00 |
|  | 23 | COVID-19, COVID-19 epidemic, Mask, Nucleic Acid, Asymptomatic Patient, Novel Coronavirus, vaccine | 0.838 | 10545.00 |
|  | 24 | COVID-19, COVID-19 epidemic, Mask, Nucleic Acid, Asymptomatic Patient, Novel Coronavirus, vaccine | 0.838 | 9438.20 |
|  | 25 | COVID-19, COVID-19 epidemic, Mask, Asymptomatic Patient, Nucleic Acid, Novel Coronavirus, vaccine | 0.838 | 8200.60 |
|  | 26 | COVID-19, COVID-19 epidemic, Mask, Asymptomatic Patient, Nucleic Acid, Novel Coronavirus, vaccine | 0.840 | 7443.60 |
|  | 27 | COVID-19, COVID-19 epidemic, Mask, Novel Coronavirus, Asymptomatic Patient, Nucleic Acid | 0.841 | 7735.80 |
|  | 28 | COVID-19, COVID-19 epidemic, Mask, Novel Coronavirus, Asymptomatic Patient, Nucleic Acid, vaccine | 0.847 | 7951.40 |
|  | 29 | COVID-19, COVID-19 epidemic, Mask, Novel Coronavirus, Asymptomatic Patient, Nucleic Acid, vaccine | 0.845 | 8538.10 |
|  | 30 | COVID-19, COVID-19 epidemic, Mask, Novel Coronavirus, Nucleic Acid, Asymptomatic Patient | 0.843 | 9660.40 |
| Fujian Province | 0 | Novel Coronavirus, COVID-19, COVID-19 epidemic, Nucleic Acid, Mask, Asymptomatic Patient, vaccine | 0.834 | 167.99 |
|  | 1 | Novel Coronavirus, COVID-19, COVID-19 epidemic, Nucleic Acid, Mask, Asymptomatic Patient, vaccine | 0.834 | 157.19 |
|  | 2 | Novel Coronavirus, COVID-19, COVID-19 epidemic, Nucleic Acid, Mask, Asymptomatic Patient, vaccine | 0.837 | 148.77 |
|  | 3 | Novel Coronavirus, COVID-19, COVID-19 epidemic, Nucleic Acid, Mask, Asymptomatic Patient, vaccine | 0.839 | 145.22 |
|  | 4 | Novel Coronavirus, COVID-19, COVID-19 epidemic, Nucleic Acid, Mask, Asymptomatic Patient, vaccine | 0.842 | 151.25 |
|  | 5 | Novel Coronavirus, COVID-19, COVID-19 epidemic, Nucleic Acid, Mask, Asymptomatic Patient, vaccine | 0.844 | 165.59 |
|  | 6 | Novel Coronavirus, COVID-19, COVID-19 epidemic, Nucleic Acid, Mask, Asymptomatic Patient, vaccine | 0.846 | 181.56 |
|  | 7 | Novel Coronavirus, COVID-19, COVID-19 epidemic, Nucleic Acid, Mask, Asymptomatic Patient, vaccine | 0.848 | 192.81 |
|  | 8 | Novel Coronavirus, COVID-19, COVID-19 epidemic, Nucleic Acid, Mask, Asymptomatic Patient, vaccine | 0.851 | 201.70 |
| Quanzhou Prefecture of Fujian Province | 0 | Novel Coronavirus, COVID-19 epidemic, COVID-19, Nucleic Acid, Asymptomatic Patient, Mask, vaccine | 0.740 | 225.57 |
|  | 1 | Novel Coronavirus, COVID-19 epidemic, COVID-19, Nucleic Acid, Asymptomatic Patient, Mask, vaccine | 0.743 | 215.89 |
|  | 2 | Novel Coronavirus, COVID-19 epidemic, COVID-19, Nucleic Acid, Asymptomatic Patient, vaccine, Mask | 0.746 | 230.03 |
|  | 3 | Novel Coronavirus, COVID-19 epidemic, COVID-19, Nucleic Acid, Asymptomatic Patient, vaccine, Mask | 0.749 | 236.28 |
|  | 4 | Novel Coronavirus, COVID-19 epidemic, COVID-19, Nucleic Acid, Asymptomatic Patient, vaccine, Mask | 0.750 | 267.52 |
|  | 5 | Novel Coronavirus, COVID-19 epidemic, COVID-19, Nucleic Acid, Asymptomatic Patient, vaccine, Mask | 0.752 | 282.04 |
|  | 6 | Novel Coronavirus, COVID-19 epidemic, COVID-19, Nucleic Acid, Asymptomatic Patient, vaccine, Mask | 0.756 | 291.06 |
|  | 7 | Novel Coronavirus, COVID-19 epidemic, COVID-19, Nucleic Acid, Asymptomatic Patient, vaccine, Mask | 0.755 | 295.75 |
|  | 8 | Novel Coronavirus, COVID-19 epidemic, Nucleic Acid, COVID-19, Asymptomatic Patient, vaccine, Mask | 0.761 | 298.82 |

AIC: Akaike information criterion.
